# Functional brain network stability reflects individual resilience during sleep deprivation

**DOI:** 10.1093/sleep/zsag201

**Published:** 2026-07-21

**Authors:** Jungwon Cha, David Negelspach, Alisa Huskey, Kathryn E R Kennedy, Jason Katz, Daniel B Forger, William D S Killgore

**Affiliations:** Social, Cognitive, and Affective Neuroscience (SCAN) Laboratory, Department of Psychiatry, University of Arizona College of Medicine - Tucson, 1501 N. Campbell Avenue, Tucson, AZ, United States; Social, Cognitive, and Affective Neuroscience (SCAN) Laboratory, Department of Psychiatry, University of Arizona College of Medicine - Tucson, 1501 N. Campbell Avenue, Tucson, AZ, United States; Social, Cognitive, and Affective Neuroscience (SCAN) Laboratory, Department of Psychiatry, University of Arizona College of Medicine - Tucson, 1501 N. Campbell Avenue, Tucson, AZ, United States; Social, Cognitive, and Affective Neuroscience (SCAN) Laboratory, Department of Psychiatry, University of Arizona College of Medicine - Tucson, 1501 N. Campbell Avenue, Tucson, AZ, United States; Social, Cognitive, and Affective Neuroscience (SCAN) Laboratory, Department of Psychiatry, University of Arizona College of Medicine - Tucson, 1501 N. Campbell Avenue, Tucson, AZ, United States; Department of Mathematics, University of Michigan, Ann Arbor, MI, United States; Gilbert S. Omenn Department of Computational Medicine and Bioinformatics, University of Michigan, Ann Arbor, MI, United States; Social, Cognitive, and Affective Neuroscience (SCAN) Laboratory, Department of Psychiatry, University of Arizona College of Medicine - Tucson, 1501 N. Campbell Avenue, Tucson, AZ, United States

**Keywords:** sleep deprivation, cognitive resilience, thalamus, visual cortex, functional connectivity, network-based statistics, nodal strength, resting-state fMRI, fatigue monitoring

## Abstract

**Study Objectives:**

Sleep deprivation is known to impair cognitive performance, but individuals differ in their ability to maintain function under sleep loss. This study examined the neural mechanisms that support such resilience by tracking changes in resting-state functional connectivity during prolonged wakefulness.

**Materials and Methods:**

Six resting-state fMRI sessions obtained during approximately the first 32 hours of a 39-hour total sleep deprivation protocol were analyzed in 16 healthy adults, enabling unusually dense longitudinal sampling of brain network dynamics during prolonged wakefulness. Functional connectivity changes were analyzed using network-based statistics as the primary approach for analyzing pairwise functional connectivity, with nodal strength used as a secondary node-level follow-up analysis in relation to a behavioral resilience index derived from psychomotor vigilance performance.

**Results:**

Two NBS-defined subnetworks, referred to here as the thalamocortical and perceptual-memory subnetworks, showed significant interactions between time awake and resilience, indicating that the trajectory of functional connectivity across prolonged wakefulness differed as a function of behavioral resilience. Participants with higher resilience showed less negative connectivity trajectories within these networks, particularly in the thalamus, globus pallidus, and visual cortex. In contrast, participants with lower resilience exhibited progressive declines in connectivity.

**Conclusion:**

The ability to withstand the cognitive effects of sleep deprivation appears to be associated with less negative connectivity trajectories within thalamocortical and perceptual-memory subnetworks. These patterns may represent state-dependent neural correlates of resilience during sleep deprivation and may be relevant to future fatigue-state monitoring.

**Statement of Significance**

People differ widely in how well they stay alert and perform when they lose sleep, but the brain basis of this resilience is still not well understood. By repeatedly measuring brain activity during extended wakefulness, we found that people who performed better also showed stronger connectivity in brain systems linked to alertness and higher-level visual processing. Those who remained more resilient kept these brain systems working together more effectively, while those who were more vulnerable showed a greater loss of coordination and poorer performance over time. These findings contribute to understanding why some people maintain performance better than others during sleep loss and may support future efforts to monitor fatigue and protect performance in high-demand settings.

## Graphical Abstract

**Graphical Abstract:**
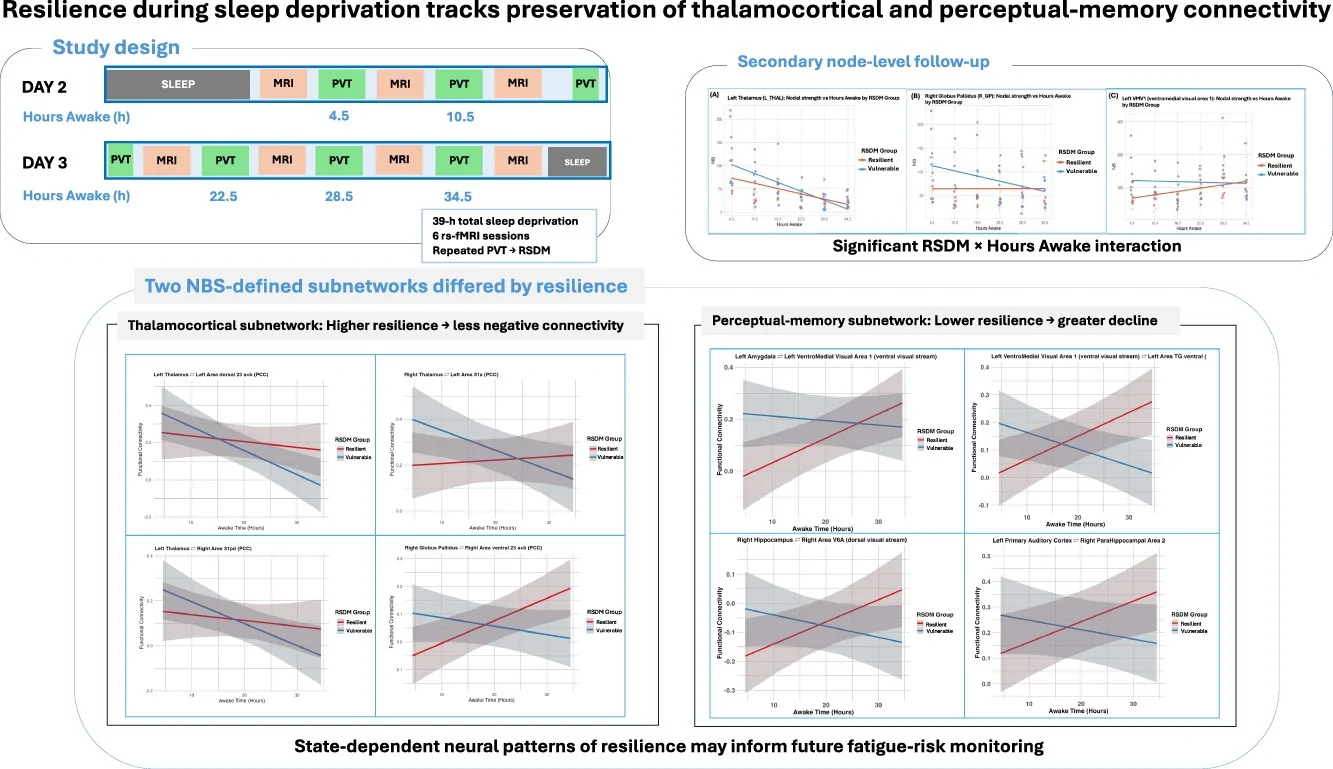

## Introduction

Sleep deprivation impairs core cognitive processes, disrupts emotion regulation, and alters brain physiology [1, 2]. Its effects are particularly evident among those in professions requiring long, uninterrupted work hours, such as medicine, transportation, and military service. In clinical settings, for example, a large U.S. cohort study found that 67.4% of medical interns reported working shifts exceeding 30 consecutive hours, often in intensive care units [3]. Similarly, in high-tempo military operations, personnel may remain awake for more than 40 hours, as extended periods without rest are frequently unavoidable [4]. Such prolonged wakefulness can slow reaction times by up to 50% and increase decision-making errors, particularly in tasks requiring sustained attention, working memory, and executive control [5–9]. However, the extent of impairment varies considerably between individuals, raising the question of why some maintain cognitive performance while others show rapid decline under equivalent sleep loss.

People differ widely in how severely performance declines during sleep deprivation [10]. We refer to this as cognitive resilience, meaning the ability to maintain performance despite severe fatigue or stress. Some individuals show marked declines in vigilance and response speed after only a few hours of sleep loss, whereas others are able to maintain relatively strong performance for much longer [11, 12]. These differences appear to be stable and trait-like, even across repeated sleep-deprivation exposures and over periods of years [13]. Even so, the underlying mechanisms remain difficult to pin down. Genetic influences may play a role in this trait-like vulnerability, including polymorphisms in genes related to circadian regulation and sleep homeostasis (e.g. PER3 and ADORA2A) [14], but they do not by themselves explain the neural changes that emerge during ongoing sleep deprivation. Likewise, evidence for demographic and other biological or psychological predictors, including age, has been mixed rather than absent, and many studies have relied on fairly restricted samples [15, 16]. For that reason, identifying the neural processes that accompany inter-individual differences during prolonged wakefulness remains an important and understudied goal [16–18].

Resilience to sleep loss can be quantified in various ways. Most common approaches have focused on either the number of attentional lapses on the psychomotor vigilance test (PVT) or a metric of response speed (i.e. 1000/RT), each yielding somewhat different classifications of resilience and vulnerability [19]. For the present study, we defined the resilience to sleep deprivation metric (RSDM), a longitudinal summary index of how well each participant preserved PVT reaction speed relative to baseline across repeated assessments during prolonged wakefulness. While baseline cognitive traits such as working memory capacity have also been linked to resilience, RSDM is derived from repeated behavioral measurements obtained during the deprivation period itself [20–22]. Baseline predictors and during-deprivation neural correlates address related but distinct questions: the former ask who may be vulnerable before sleep loss begins, whereas the latter address how resilience is expressed as sleep deprivation unfolds.

While behavioral metrics such as RSDM provide a powerful means for assessing individual resilience, they do not directly reveal the underlying neural correlates of vulnerability or stability. To bridge this gap, neuroimaging studies have explored resting-state functional MRI (rs-fMRI) connectivity as a window into brain network dynamics during sleep deprivation. Prior neuroimaging work has shown that prolonged wakefulness alters large-scale brain function, including resting-state connectivity within the default mode network (DMN), Salience Network (SN), and frontoparietal systems [23–25].

Importantly, not all prior neuroimaging studies have addressed the same question. For example, Yeo and colleagues showed that resting-state functional connectivity acquired during rested wakefulness can predict later vulnerability to sleep deprivation [26]. That baseline-prediction approach differs from the present study, which does not test vulnerability before deprivation begins, but instead examines how functional connectivity changes during ongoing sleep deprivation covary with behavioral resilience. Accordingly, our findings should be interpreted as neural correlates expressed during the sleep-loss state rather than as pre-deprivation predictors.

Recent studies have applied graph-theoretical analyses to rs-fMRI to examine how sleep loss alters large-scale network topology. Sleep deprivation has been linked to reduced global efficiency and disrupted modular organization within the DMN, SN, thalamocortical, and sensorimotor systems [27–29]. Among node-level metrics, nodal strength (NS), defined as the weighted degree of a node, provides a simple summary of the overall strength of a region’s functional connectivity with the rest of the network. Although NS has been widely used in graph-theoretical analyses of brain networks, it has rarely been examined specifically in the context of sleep deprivation [30–32]. In this framework, decreased NS would reflect functional disconnection with extended wakefulness, whereas enhanced NS may signal neural resilience. This approach also builds on structural findings linking frontoparietal and triple-network white-matter integrity to resistance against sleep loss [20, 33].

Complementary to nodal metrics, network-based statistics (NBS) allows detection of subnetworks in which pairwise functional connectivity is modulated by behavioral or experimental factors. Unlike global graph measures, NBS identifies connected sets of pairwise functional connections associated with behavioral or experimental variables.

Prior research has shown that greater daytime sleepiness is associated with reduced thalamocortical functional connectivity, suggesting that disruptions in these circuits may underlie early resilience deficits during sleep loss [34]. Based on this and related prior work, we hypothesized that individuals with higher RSDM would show less negative connectivity trajectories in thalamic, visual, and salience-related regions, whereas those with lower RSDM would show progressive declines. To test these hypotheses, we modeled RSDM as a continuous index of resilience and examined its interaction with hours awake on resting-state functional connectivity in a longitudinal within-subject fMRI dataset. As a secondary, node-level follow-up, we also examined nodal strength within regions implicated by the primary NBS results in order to characterize how the overall connectivity of these nodes changed across prolonged wakefulness. This approach allowed us to relate progressive network changes during prolonged wakefulness to individual differences in behavioral resilience. Our goal was to identify network-level neural correlates of cognitive resilience expressed during sleep deprivation and to further characterize the node-level topology of regions participating in the identified subnetworks.

## Materials and methods

### Participants and study design

Twenty-one healthy adults were recruited for a controlled 4-day, in-laboratory protocol consisting of a baseline night of sleep, approximately 39 hours of total sleep deprivation (TSD), and a recovery night of sleep (Figure 1A). Participants were continuously monitored in the same in-laboratory suites and were maintained under the same facility-level environmental controls and protocol schedule throughout the study, including controlled lighting, temperature regulation, minimized environmental noise, fixed meal timing, restricted activity, and scheduled testing blocks.

**Figure 1:**
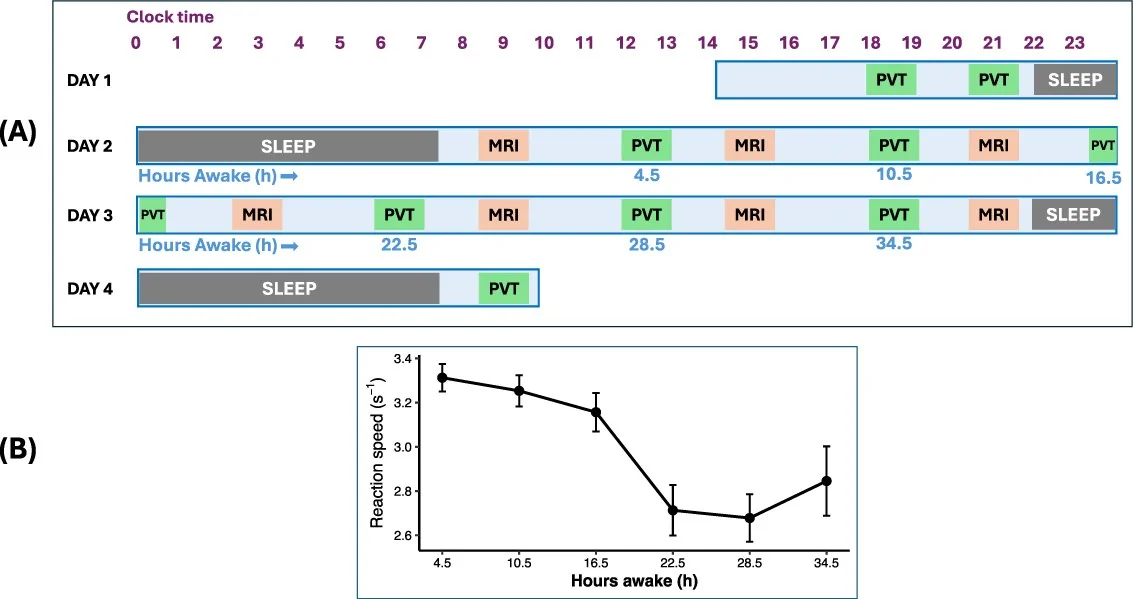
Experimental protocol and behavioral trajectory during sleep deprivation. (A) Schematic of the experimental protocol across four consecutive days. Participants underwent repeated MRI sessions and psychomotor vigilance task (PVT) assessments during a period of total sleep deprivation. MRI sessions were conducted approximately every 6 hours, and PVT assessments were administered approximately 2.5 hours after sessions 1–6. Time points are expressed as hours awake, with corresponding clock times indicated to illustrate circadian phase. Sleep opportunities are shown where applicable. Behavioral and imaging analyses were restricted to sessions 1–6 to preserve a more consistent temporal relationship between MRI and PVT measures, as the PVT associated with session 7 occurred more than 6 hours after scanning. (B) Group-level changes in PVT reaction speed as a function of hours awake. Reaction speed was calculated as the reciprocal of mean reaction time (1000/RT), such that higher values indicate faster responding. Data points represent group means, and error bars indicate the standard error of the mean (SEM). Reaction speed progressively declined with increasing time awake, followed by a modest rebound at later time points, which may reflect circadian modulation rather than full recovery.

During the TSD period, seven MRI sessions were scheduled at approximately 6-hour intervals, spanning approximately 32 hours from the first post-baseline scan to the final scan. Although neuroimaging was acquired at all seven time points, only sessions 1–6 were included in the present imaging-behavior analyses. The final session was excluded because the corresponding PVT occurred more than 6 hours after scanning, whereas the PVT assessments corresponding to sessions 1–6 occurred approximately 2.5 hours after imaging. Because vigilance performance changes progressively with increasing time awake, the longer delay for session 7 was judged too large for the present imaging-behavior models, whereas the remaining sessions were retained with the temporal-offset limitation acknowledged below and in the Limitations section. This exclusion applied only to the analytic stage of the imaging-behavior models and did not affect the predefined participant inclusion criteria based on completion of the full acquisition protocol.

Behavioral testing continued through the full 39-hour TSD period. Five participants were excluded from imaging analyses based on predefined protocol-completion criteria. Four participants lacked adequate resting-state fMRI coverage across the full acquisition protocol, defined as either having no usable rs-fMRI data at any session or missing one or more of the scheduled MRI sessions. One additional participant discontinued the protocol before completion. The final analytic sample comprised 16 participants with usable neuroimaging data from sessions 1-6. Demographic and behavioral data for the final sample are presented in Table 1.

**Table 1:** Demographic and behavioral data. Baseline lapses refers to the number of lapses (>500 ms response time) on the PVT during the first session

	**Mean**	**SD**	**Range**
**Demographics**			
Age (years)	23.6	4.6	18–36
Gender (female/male)	8 / 8		
**Psychomotor vigilance test**			
RSDM (%)	83.5	11.3	46.8–96.8
Baseline lapses	1.1	1.2	0–4

#### Computation of hours awake and circadian terms

Hours Awake was defined as clock time (in hours) elapsed since scheduled wake time (07:30) on Day 2, accumulated without sleep until recovery. Scan start times were mapped to Hours Awake for all acquired MRI sessions. To approximate circadian influences, we included sinusoidal terms sin(2π·CT/24) and cos(2π·CT/24) in the statistical models, where CT is clock time in hours. Including both sine and cosine components allows the model to capture a phase-shifted circadian rhythm without specifying the phase parameter (ϕ) directly, since a linear combination of sine and cosine terms is mathematically equivalent to a phase-shifted sinusoid (i.e. Asin(ωt) + Bcos(ωt) = Rsin(ωt + ϕ)).

### Psychomotor vigilance task

PVT scores for the present analyses were derived from a standard 10-minute PVT with random inter-stimulus intervals of 2–10 seconds. The PVT is highly sensitive to sleep loss and shows progressive declines in vigilance performance with increasing time awake during sleep deprivation [6–8, 35] (Figure 1B).

### Behavioral resilience to sleep deprivation metric

The RSDM was calculated based on participants’ performance on the PVT. In the present protocol, the PVT was administered approximately 2.5 hours after each fMRI scan as part of a standardized behavioral testing schedule that was repeated throughout the sleep deprivation period. Behavioral assessments were organized into fixed testing blocks rather than being performed immediately before or after each scan, in order to maintain a consistent protocol across the 39-hour deprivation period. Although the PVT was not administered directly adjacent to each MRI session, the delay was relatively consistent across sessions 1–6; therefore, these PVT assessments were used as the corresponding behavioral measures for the imaging-behavior models, while acknowledging that the temporal offset may reduce the precision of brain-behavior alignment, as discussed in the Limitations. RSDM was calculated to summarize the degree of cognitive performance preservation across increasing sleep pressure. Specifically, RSDM was computed as:


$$ \mathbf{RSDM}=\frac{\ \mathbf{mean}\ \mathbf{reaction}\ \mathbf{speed}\ \mathbf{during}\ \mathbf{sessions}\ \mathbf{4}-\mathbf{6}}{\mathbf{mean}\ \mathbf{reaction}\ \mathbf{speed}\ \mathbf{during}\ \mathbf{sessions}\ \mathbf{1}-\mathbf{3}}\times \mathbf{1}\mathbf{00}\% $$

Higher RSDM values indicate greater resilience to the detrimental effects of sleep loss on vigilance. An RSDM value of 100% indicates that mean reaction speed was preserved at the same level in sessions 4–6 as in sessions 1–3. Values below 100% indicate a decline in reaction speed during the later portion of sleep deprivation relative to baseline, whereas values above 100% indicate preserved or improved reaction speed relative to baseline.

### MRI acquisition and preprocessing

Neuroimaging was performed on a Siemens 3T Skyra. Seven sessions were scheduled at ~6-hour intervals (08:30, 14:30, 20:30 on day 2; 02:30, 08:30, 14:30, 19:30 on day 3). Although all seven MRI sessions were acquired, only sessions 1–6 were included in the imaging-behavior analyses, as described above. Structural images used a T1-weighted MPRAGE (1.0-mm isotropic; TR = 2100 ms; TE = 2.33 ms; 176 slices; FOV = 256 mm). Resting-state fMRI used gradient-echo EPI (2.0-mm isotropic; TR = 2000 ms; TE = 36 ms; 60 slices; 180 volumes; FOV = 240 mm); participants fixated on a central cross with eyes open and were instructed to minimize motion. Analyses in this report used rs-fMRI from sessions 1-6. Preprocessing was performed using AFNI (Analysis of Functional NeuroImages) tools [36], following the standard processing framework implemented in afni_proc.py, although the pipeline was executed manually rather than through the wrapper itself. Processing steps included slice-timing correction and motion correction using 3dvolreg, followed by alignment of functional images to each participant’s anatomical T1-weighted image. Anatomical images were nonlinearly normalized to the MNI152 template using AFNI’s 3dQwarp, following the nonlinear alignment approach described by Saad et al. [37]. The EPI-to-anatomical and anatomical-to-MNI transformations were concatenated and applied in a single step to the functional data to avoid multiple interpolations. Nuisance regression and temporal filtering were performed simultaneously within a general linear model framework. Regressors included six motion parameters along with mean signals from white matter and cerebrospinal fluid. No explicit motion censoring was applied; instead, motion effects were accounted for through inclusion of motion parameters in the regression model. Bandpass filtering (0.01–0.1 Hz) was applied within this model to reduce physiological noise in the BOLD signal.

### Region of interest definition and functional connectivity estimation

Time series data were extracted from 375 regions of interest (ROIs) using the Human Connectome Project (HCP) cortical atlas [38], along with the Harvard-Oxford subcortical atlas [39]. For each session, Pearson correlation coefficients were calculated between ROI pairs, yielding a 375 × 375 weighted, undirected functional connectivity matrix based on Fisher z-transformed Pearson correlations. These correlation values were retained as signed values (i.e. positive and negative correlations were preserved) and were Fisher z-transformed for subsequent analyses. No absolute-value transformation was applied. ROI labels follow the HCP cortical and Harvard-Oxford subcortical atlases. Abbreviations are used in figures for clarity, and full ROI names are provided in Table 2.

**Table 2:** Grouped summary of NBS-defined subnetwork edges

**Network **	**ROI 1**	**ROI 2**	**Functional connectivity**
Thalamocortical subnetwork	L_THAL / R_THAL	L_d23ab / L_31pv / L_31a / R_d23ab / R_31pv / R_31pd / R_31a / R_v23ab (PCC)	Thalamus—posterior cingulate cortex
	L_THAL / R_THAL	R_POS2 (retrosplenial)	Thalamus—retrosplenial cortex
	R_THAL	L_5mv (SPL)	Thalamus—superior parietal lobule
	L_THAL	R_9p / R_10d (prefrontal)	Thalamus—prefrontal cortex
	L_GP / R_GP	R_POS1 / R_v23ab / R_7m	Pallidum—posterior cingulate/superior parietal regions
	L_PreS / R_PreS	R_31pd / R_7m / L_31pv / L_31pd	Presubiculum—posterior cingulate/superior parietal regions
	L_TA2 / L_PI	R_POS1	Planum polare/parainsular—retrosplenial
	R_POS1	R_Pir	Retrosplenial cortex—piriform cortex
**Network**	**ROI 1**	**ROI 2**	**Functional connectivity**
Perceptual-memory subnetwork	L_AMYG / R_AMYG / L_Pir	L_VMV1	Amygdala/piriform—ventromedial visual cortex
	L_43	L_VMV1	Area 43—ventromedial visual cortex
	L_4 / L_3b / L_3a / L_43	L_VVC	Somatomotor/opercular—ventral visual complex
	L_VMV1 / L_ProS	L_TGv / R_TGv	Ventral visual/prostriate—temporal pole
	L_VVC	L_A4	Ventral visual complex—auditory area 4
	L_HIPP	R_4	Hippocampus—primary motor cortex
	L_A1 / L_PBelt / L_LBelt / L_A4	R_PHA2	Primary/belt auditory—parahippocampal cortex
	R_HIPP / R_AMYG	R_V6A	Amygdala/hippocampus—dorsal visual cortex
	L_3b / L_1 / L_6d / L_PFcm / R_4 / R_3b / R_1	R_TF	Somatosensory/motor/opercular—temporal fusiform cortex
	R_TF	R_LBelt	Temporal fusiform cortex—auditory belt
	L_HIPP	R_VMV1	Hippocampus—ventromedial visual cortex
	R_PHA2	R_A4	Parahippocampal cortex—auditory area 4
	L_43	L_ProS	Area 43—prostriate cortex

This table summarizes the rs-fMRI edges contributing to the significant RSDM × Hours Awake interaction identified by the Network-Based Statistics (NBS) analysis (p_FWE < 0.05). Rows represent grouped anatomical connectivity motifs and may therefore summarize multiple individual edges within each NBS-defined component. Edges are grouped into recurring anatomical-functional motifs within the two NBS-defined subnetworks: the thalamocortical subnetwork and the perceptual-memory subnetwork. ROI labels follow the HCP cortical atlas and Harvard-Oxford subcortical labels; hemisphere is denoted L/R. All significant edges are included. Abbreviations (with full names): THAL, thalamus; PCC, posterior cingulate cortex (subdivisions: d23ab = dorsal 23 a + b; v23ab = ventral 23 a + b; 31 pv = ventral 31p; 31pd = dorsal 31p; 31a = area 31a); POS1/2, retrosplenial cortex (parieto-occipital sulcus areas 1/2); SPL, superior parietal lobule (5mv = area 5 m ventral; 7 m = area 7 m); 9p, posterior area 9 (dlPFC); 10d, frontopolar cortex; GP, globus pallidus; PreS, presubiculum; PI, parainsular area; TA2, planum polare (area TA2); Pir, piriform cortex (olfactory cortex); ProS, prostriate cortex (early visual); VMV1, ventromedial visual area 1; VVC, ventral visual complex; V6A, dorsal visual area V6A; TGv, temporal pole (area TG ventral); TF, temporal fusiform cortex; HIPP, hippocampus; PHA2, parahippocampal area 2; AMYG, amygdala; A1, primary auditory cortex; PBelt/LBelt, auditory belt areas; A4, auditory area 4; Areas 1/3a/3b, primary somatosensory cortex; Area 4, primary motor cortex; 6d, dorsal premotor area; PFcm, area PFcm (posterior operculum); 43, area 43 (posterior operculum). Functional descriptions were standardized across repeated ROIs to ensure consistent terminology throughout the table. Each recurring ROI was assigned a single canonical anatomical-functional label used consistently across all entries

### Network-based statistics

Network-based statistics [40] were used to identify subnetworks where resting-state functional connectivity (rsFC) was significantly modulated by the interaction between RSDM and Hours Awake. In the present framework, nodes correspond to the 375 ROIs from the HCP and Harvard-Oxford atlases, and edges represent the pairwise functional connectivity between ROI time series. Each edge value was defined as the signed Fisher z-transformed Pearson correlation between two ROIs during a given fMRI session. Thus, NBS analyses test whether the strength of a specific pairwise functional connection varies as a function of behavioral resilience (RSDM) and hours awake. The general linear model was implemented at the edge level, with the following formulation:


\begin{align*} \boldsymbol{rsFC}\sim&\, \boldsymbol{RSDM}\ast \boldsymbol{Hours}\_\boldsymbol{Awake}+\boldsymbol{Baseline}\_\boldsymbol{Lapse}+\boldsymbol{\sin}\_\boldsymbol{ct}\nonumber\\&+\boldsymbol{\cos}\_\boldsymbol{ct}+\left(\mathbf{1}\ |\ \boldsymbol{Subject}\right) \end{align*}

The model was constructed to test whether the strength of each rsFC between ROI pairs varied as a function of the interaction between RSDM and accumulated wakefulness. The term Baseline_Lapse represents the number of lapses during session 1 and was included to account for trait-level differences in vigilance capacity prior to sleep deprivation. Although the mixed-effects model includes subject-level random intercepts, the Baseline_Lapse covariate was included to capture individual variability in well-rested vigilance performance that might influence resilience trajectories during subsequent sleep deprivation (i.e. when well rested). The terms $\mathit{\sin}\_ ct$ and $\mathit{\cos}\_ ct$ refer to the sine and cosine transformations of the time-of-day (clock time (CT)) as sin(2π·CT/24) and cos(2π·CT/24) respectively and were included to model circadian rhythm influences on brain connectivity [41]. Finally, a random intercept for each participant (1 | Subject) was included in the model to accommodate the repeated-measures structure of the data, where multiple fMRI sessions were nested within individuals. A primary t-threshold of 3.1 was used to identify suprathreshold connections. The resulting clusters of functionally connected edges were subjected to permutation testing (5000 iterations) to derive cluster-level *p*-values with familywise error (FWE) correction. All follow-up graph-theoretical analyses (e.g. NS) were restricted to the union of ROIs participating in NBS-significant components. Because these node-level analyses were conducted within regions identified by the primary NBS results, they were treated as secondary, post hoc characterizations of the NBS-defined subnetworks rather than as independent confirmatory tests. Multiple-comparison control for these follow-up analyses was therefore confined to this ROI set. For visualization in Figures only (not for statistical analyses), participants were stratified into high and low RSDM groups using the top 40% (n = 6) and bottom 40% (n = 6) of the RSDM distribution within the final sample of 16 participants. All statistical models treated RSDM and Hours Awake as continuous variables. Visualization of NBS results was performed for interpretability only and does not constitute an additional inferential step. The labels used for the identified subnetworks in the Results section are descriptive summaries of the predominant anatomical regions involved and are not intended to imply predefined functional network identities.

### Nodal strength analysis

As a secondary, node-level follow-up to the primary NBS analysis, we used the GTG toolbox [42] to compute nodal strength (NS) from each 375 × 375 connectivity matrix for ROIs participating in the NBS-defined subnetworks. NS, defined as the weighted degree, was calculated as the sum of Fisher-z-transformed functional connectivity values between each ROI and all others in the network. In the present study, lower NS was interpreted as reduced overall functional coupling of a node with the rest of the network, whereas stable or higher NS indicated relatively preserved connectivity across prolonged wakefulness. We selected NS as a simple node-level summary of each region’s aggregate functional connectivity with other regions within the network. In the present study, NS was used to characterize whether nodes participating in the NBS-defined subnetworks also showed changes in overall connectivity across prolonged wakefulness.

We examined how NS varied across sessions as a function of behavioral resilience and time awake. For each ROI, we implemented a linear mixed-effects model in R:


\begin{align*} \boldsymbol{NS}\sim&\, \boldsymbol{RSDM}\ast \boldsymbol{Hours}\_\boldsymbol{Awake}+\boldsymbol{Baseline}\_\boldsymbol{Lapse}+\boldsymbol{\sin}\_\boldsymbol{ct}\\&+\boldsymbol{\cos}\_\boldsymbol{ct}+\left(\mathbf{1}\ |\ \boldsymbol{Subject}\right) \end{align*}

The covariate and transformations matched those in the NBS model. The interaction term assessed whether the trajectory of NS with increasing time awake depended on behavioral resilience. A subject-level random intercept accounted for repeated measurements. Statistical inference for NS models was confined to the union of ROIs from NBS-positive components, and multiple comparisons were controlled using false discovery rate (FDR) across this ROI set. Because these ROIs were defined from the primary NBS results, the NS analysis was interpreted as a post hoc node-level characterization of the identified subnetworks rather than as an independent replication. This restricted ROI set was not selected a priori and therefore should not be interpreted as an a priori strategy for reducing the multiple-testing burden, but rather as a post hoc characterization of nodes participating in the NBS-defined subnetworks. Statistical analyses were performed in R (version 4.3.1) using the *lme4* and *lmerTest* packages for mixed-effects modeling.

## Results

### Network-based statistics: Subnetworks showing RSDM × hours awake interactions

NBS revealed two subnetworks in which the relationship between resting-state functional connectivity and hours awake depended on behavioral resilience, reflected in a significant RSDM × Hours Awake interaction. For descriptive convenience, these NBS-defined subnetworks are referred to here as the thalamocortical subnetwork (Net1; 27 edges, 24 ROIs, p_FWE < 0.05) and the perceptual-memory subnetwork (Net2; 31 edges, 30 ROIs, p_FWE < 0.05), totaling 54 unique ROIs. Each label refers to the full NBS-identified connected component detected in the present data, rather than to a predefined canonical network or to a subset of a larger named network.

For visualization (Figures 2 and 3), we displayed group-mean Fisher z-transformed connectivity values for NBS-defined edges after stratifying participants into the top 40% (Resilient) and bottom 40% (Vulnerable) of the RSDM distribution for display only. Brain renderings overlay these group-mean connectivity values on the NBS topology using a diverging scale centered at 0 with a fixed range of −0.15 to +0.15. Warmer colors indicate higher Fisher z-transformed connectivity values and cooler colors indicate lower values. Conceptually, an edge represents the connection between two ROIs, while the edge weight reflects the strength of synchronous BOLD fluctuations between their time series. Panel C in each figure shows representative edge-level connectivity trajectories across hours awake for high and low RSDM groups, illustrating how functional connectivity changes with increasing wakefulness. These visualizations are descriptive only and should not be interpreted as independent statistical evidence beyond the model-based results. Formal statistical inference is provided by the mixed-effects models and permutation-based NBS results reported above. For completeness, descriptive PVT reaction-speed trajectories for the same high and low RSDM groups are provided in Figure S1, and the full unthresholded interaction-statistic matrix is provided in Figure S2.

**Figure 2:**
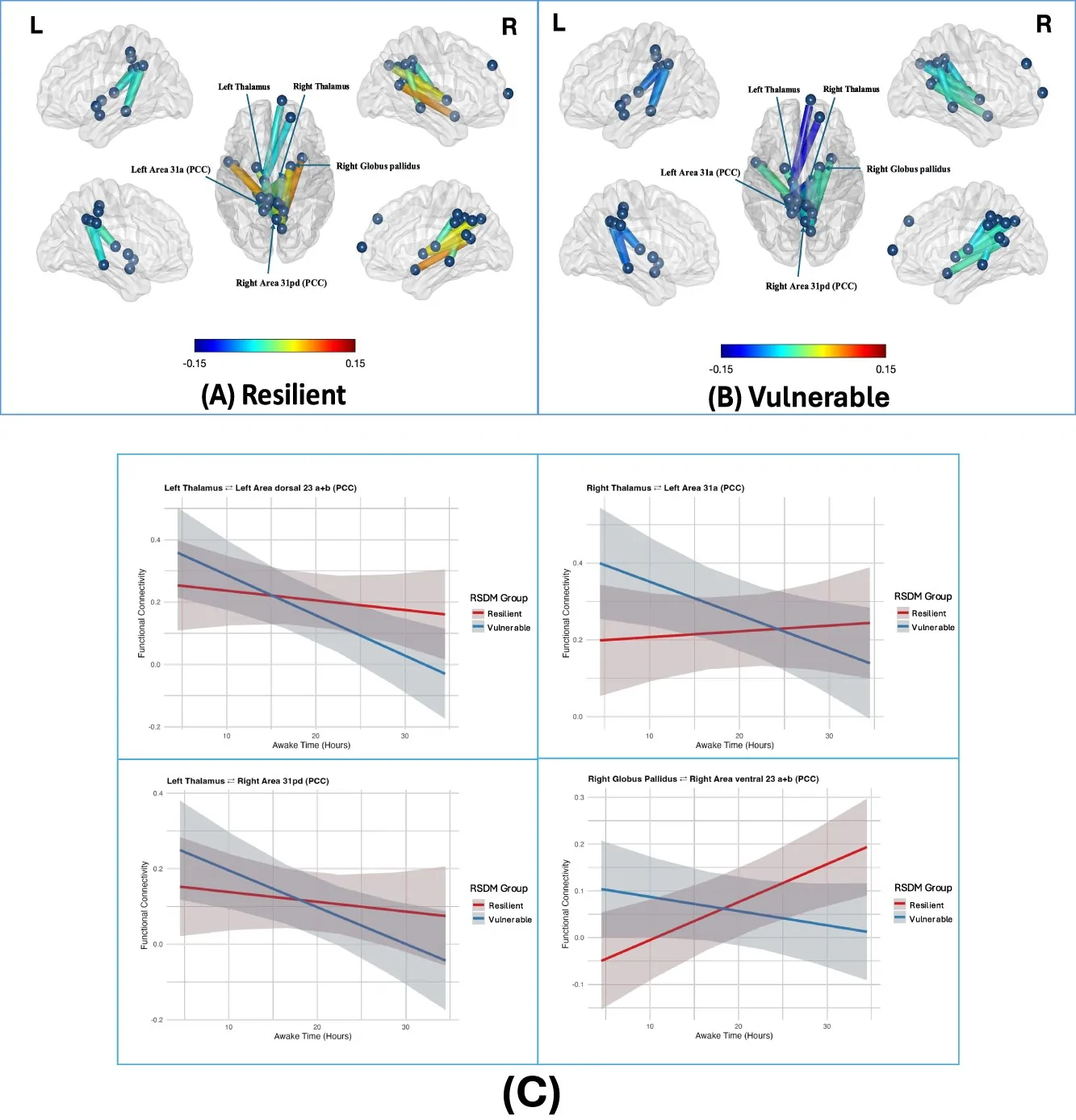
Thalamocortical subnetwork during prolonged wakefulness. (A) Group-mean edge-wise Fisher z-transformed rs-fMRI connectivity values for the high RSDM group, overlaid on the NBS-defined thalamocortical subnetwork topology. (B) Corresponding group-mean edge-wise Fisher z-transformed rs-fMRI connectivity values for the low RSDM group. Panels A and B are shown for visualization only; edge-wise connectivity values are displayed using the same numerical scale, ranging from −0.15 to +0.15. (C) Representative rs-fMRI connectivity trajectories across hours awake for high and low RSDM groups at selected edges within the thalamocortical subnetwork. Lines represent group-mean regression fits for visualization only. Full ROI names appear in Table 2. These visualizations are descriptive and were not used for statistical inference.

**Figure 3:**
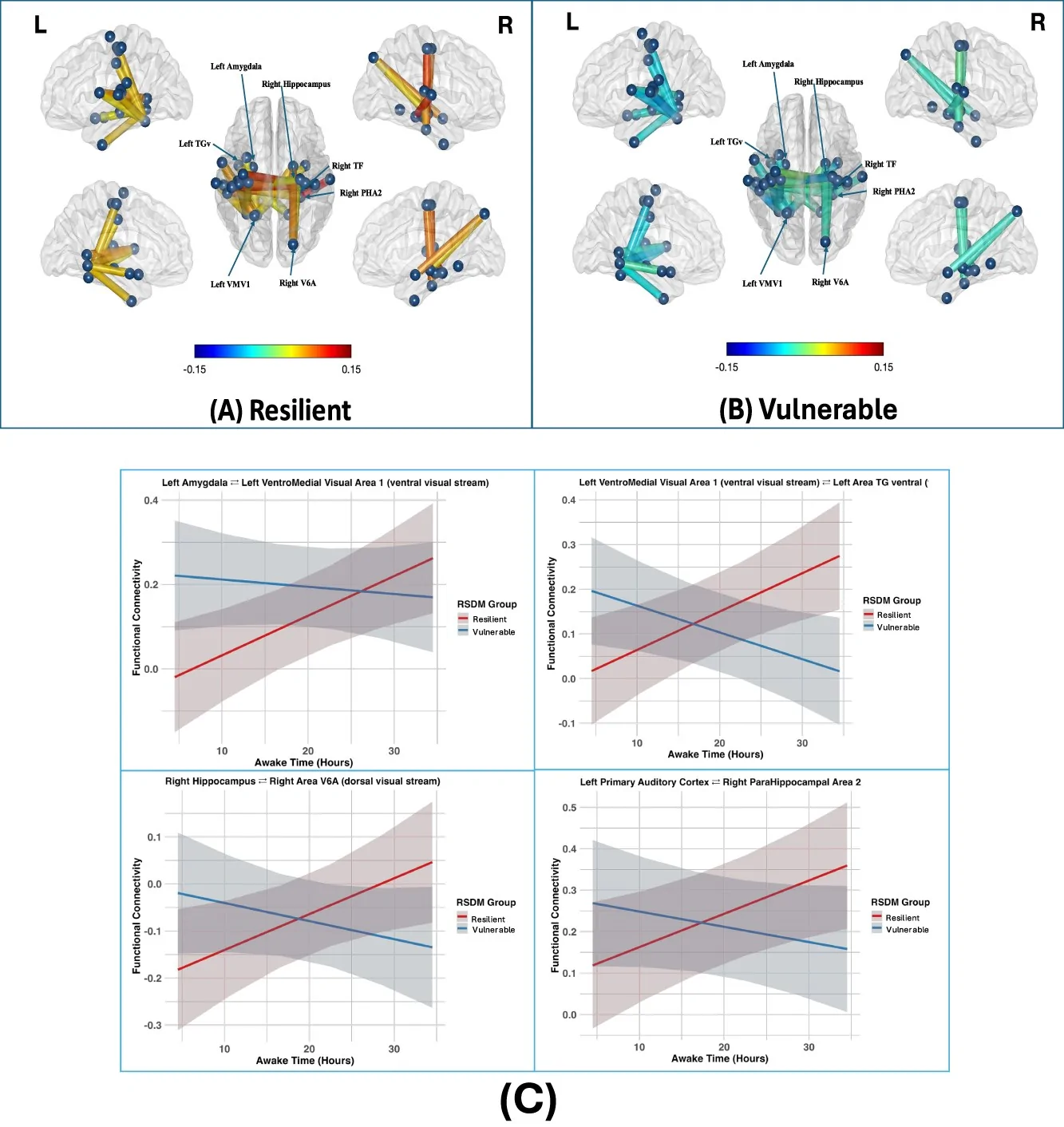
Perceptual-memory subnetwork during prolonged wakefulness. (A) Group-mean edge-wise Fisher z-transformed rs-fMRI connectivity values for the high RSDM group, overlaid on the NBS-defined perceptual-memory subnetwork topology. (B) Corresponding group-mean edge-wise Fisher z-transformed rs-fMRI connectivity values for the low RSDM group. Panels A and B are shown for visualization only; connectivity values are displayed on the same numerical scale as in Figure 2. (C) Representative rs-fMRI connectivity trajectories across hours awake for high and low RSDM groups at selected edges within the perceptual-memory subnetwork. Lines represent group-mean regression fits for visualization only. Full ROI names appear in Table 2. These visualizations are descriptive and were not used for statistical inference.

### Subnetwork 1: Thalamocortical subnetwork (Net1)

In the Thalamocortical subnetwork, the group-mean connectivity values overlaid on the NBS topology are shown in Figure 2A (high RSDM) and Figure 2B (Low RSDM), with representative edge-level connectivity trajectories illustrated in Figure 2C. Significant edges linked bilateral thalamus and globus pallidus with posterior cingulate and retrosplenial subdivisions, with additional projections to superior parietal and prefrontal regions. For descriptive visualization only, the representative edge-level trajectories in Figure 2C suggested that participants with relatively higher RSDM tended to show less negative slopes along the thalamus-PCC axis, whereas those with relatively lower RSDM showed declining connectivity across hours of sustained wakefulness. Representative edges were selected to illustrate the principal anatomical motifs of the subnetwork, particularly thalamic and pallidal connections with posterior cingulate and retrosplenial regions; these include L_THAL-L_d23ab, L_THAL-R_31pd, L_THAL-R_POS2, and R_GP-R_v23ab. Full edge lists and ROI names are provided in Table 2.

### Subnetwork 2: Perceptual-memory subnetwork (Net2)

In the Perceptual-Memory subnetwork, the group-mean connectivity values overlaid on the NBS topology are shown in Figure 3A (high RSDM) and Figure 3B (Low RSDM), with representative edge-level connectivity trajectories illustrated in Figure 3C. Significant edges coupled ventral and dorsal visual areas with medial temporal and auditory regions, including hippocampus, amygdala, PHA2, TGv, and TF. For descriptive visualization only, the representative edge-level trajectories in Figure 3C suggested that participants with relatively higher RSDM showed attenuated declines or modest increases in visual-memory and limbic-visual links, whereas those with relatively lower RSDM showed more broadly negative slopes across hours of sustained wakefulness. Representative edges were selected to illustrate the principal visual-limbic, visual-temporal, and auditory-parahippocampal motifs of the subnetwork; these include L_AMYG-L_VMV1, L_VMV1-L_TGv, R_HIPP-R_V6A, and L_A1-R_PHA2. See Table 2 for the complete set. Positive slopes indicate increasing connectivity with prolonged wakefulness and negative slopes indicate decreasing connectivity.

### Secondary node-level follow-up: Nodal strength within NBS-defined subnetworks

As a secondary node-level follow-up, NS was examined in the 54 ROIs participating in the NBS-defined subnetworks to characterize how overall connectivity of these nodes varied as a function of the RSDM × Hours Awake interaction. Mixed-effects modeling revealed that three nodes demonstrated significant interaction effects after FDR correction.

Within this secondary follow-up analysis, node-level models revealed significant RSDM × Hours Awake interactions for left thalamus, right globus pallidus, and left VMV1 (Figure 4 and Table 3). For descriptive visualization only, participants with relatively lower RSDM showed steeper declines in nodal strength over time in subcortical regions, whereas those with relatively higher RSDM showed less steep trajectories or relatively preserved nodal strength. Visual cortex showed relatively preserved nodal strength or a less negative trajectory in the high RSDM group, consistent with relatively preserved node-level connectivity within the identified subnetworks. Interaction plots for all significant ROIs are provided in Figure 4. High and low RSDM participants are highlighted separately for visualization, with the middle 20% shown as gray points; regression lines are included only to illustrate the direction of the continuous interaction and do not represent separate group-based statistical tests.

**Figure 4:**
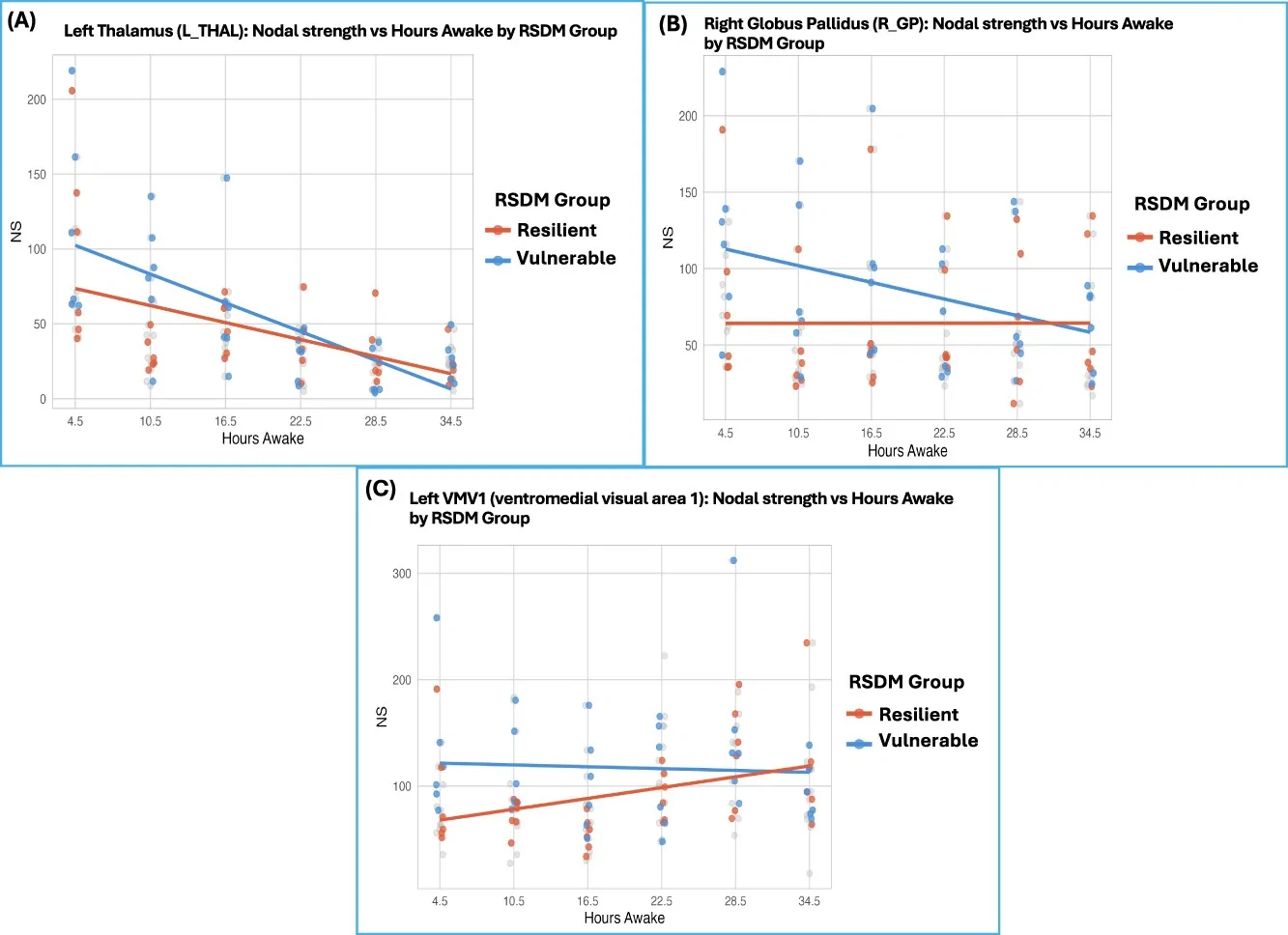
Nodal-strength (NS) trajectories within NBS-defined subnetworks for all significant follow-up ROIs. For all significant ROIs, nodal strength is plotted against hours awake. Participants were stratified into resilient/high RSDM (top 40%, n = 6) and vulnerable/low RSDM (bottom 40%, n = 6) groups for visualization purposes only; the remaining middle 20% (n = 4) are shown as gray points. All statistical analyses treated RSDM as a continuous variable in mixed-effects models. Lines show ordinary least-squares fits for visualization only and do not represent group-based statistical comparisons reported in the text. Horizontal jitter (width = 0.3 session units) was applied to all data points. All 16 participants contribute data at each of the six sessions (96 total observations per panel), confirming that no data are missing. Full statistics and adjusted *p*-values appear in Table 3.

**Table 3:** Secondary follow-up ROI-level nodal strength (NS) results within NBS-defined subnetworks

**ROI label**	**Full name**	**β (RSDM × Hours_Awake)**	**95% CI**	**p_FDR**
L_THAL	Left thalamus	0.0804	[0.0241, 0.1368]	0.0250
R_GP	Right globus pallidus	0.0771	[0.0212, 0.1330]	0.0321
L_VMV1	Left ventromedial visual area 1	0.1011	[0.0228, 0.1794]	0.0484

β is the fixed-effect interaction (RSDM × Hours_Awake). It reflects how the slope of NS across hours awake changes as a function of RSDM. A positive β indicates that the trajectory of NS becomes less negative or more positive as RSDM increases; thus, both groups may show declining slopes, but the decline is less steep at higher levels of RSDM. 95% confidence intervals (CIs) indicate the estimated range of the true effect. p_FDR values are adjusted across the NBS ROI set. ROI labels follow the HCP cortical and Harvard-Oxford subcortical atlases. L/R denotes hemisphere

The summary of statistically significant ROI-level results is provided in Table 3, including beta coefficients, and FDR-adjusted *p*-values for the RSDM × Hours Awake interaction term.

## Discussion

This study demonstrates that cognitive resilience to acute sleep deprivation is associated with the preservation of connectivity within two NBS-defined subnetworks involving thalamic, posterior cortical, and perceptual-memory-related regions. In the primary edge-level NBS analysis, higher RSDM was associated with relatively less negative trajectories within these systems, whereas lower RSDM scores were linked to more pronounced reductions in rsFC across prolonged wakefulness. Secondary node-level analyses of nodal strength within the NBS-defined subnetworks showed a similar pattern in selected regions, including thalamic, pallidal, and visual nodes. The accompanying visualizations are intended to aid interpretation of these effects and do not represent independent statistical tests beyond the reported models.

These findings suggest that resilience to sleep deprivation is associated with relatively preserved connectivity in subcortical and cortical regions implicated in vigilance and attention [16, 17, 21, 35]. This interpretation is consistent with the role of the PVT as a sensitive behavioral measure of vigilance and attentional stability during sleep deprivation [21, 35]. Given that the PVT primarily indexes sustained attention and vigilance, the observed network patterns likely reflect neural systems supporting these cognitive processes under prolonged wakefulness.

Two NBS-defined subnetworks emerged from the analysis. The subnetwork referred to here as the thalamocortical subnetwork included bilateral thalamus, globus pallidus, PCC, and retrosplenial regions, with extensions into superior parietal and prefrontal areas. The PCC and retrosplenial cortex are core hubs of the DMN [43]. This arrangement may link subcortical arousal systems with large-scale cortical states [34, 44–47]. Consistent with this interpretation, greater daytime sleepiness has been associated with reduced thalamocortical connectivity at rest, particularly with sensorimotor and ventromedial prefrontal regions [34]. In high-RSDM individuals, connections between the thalamus and PCC or retrosplenial cortex showed less negative trajectories, potentially supporting internal-model stability and efficient information flow. In low-RSDM individuals, these connections declined, suggesting reduced thalamic gating and weaker integration of sensorimotor and cognitive processes [46], in line with prior reports of thalamocortical decoupling accompanying greater sleepiness.

The subnetwork referred to here as the perceptual-memory subnetwork included VMV1, presubiculum, parieto-occipital sulcus, and medial temporal regions, consistent with the posterior medial memory network [48–51]. This system brings together scene-related visual input with contextual and memory information, supporting spatial orientation and the recall of past events. Consistent with prior work by Chee and colleagues, sleep deprivation weakens top-down biasing of activation in ventral visual cortex and reduces connectivity between parietal control regions and scene-selective cortex, and even slows rapid picture processing in higher visual areas [52, 53]. Moreover, prior work by Yeo and colleagues showed that baseline functional connectivity during rested wakefulness can predict vulnerability to sleep deprivation, implicating visual and attention networks among the discriminative features [26]. In high-RSDM individuals, connectivity within these regions showed attenuated decline across prolonged wakefulness. Because these connections are involved in spatial and contextual processing, their preservation may help mitigate the attention and memory difficulties that typically emerge with prolonged wakefulness [54].

The NS findings provide a secondary node-level characterization of the NBS-defined subnetworks, suggesting that some of the regions implicated at the edge level also showed changes in their overall connectivity across prolonged wakefulness [30–32]. In this framework, NBS identified subnetworks showing significant edge-level modulation, whereas the follow-up NS analysis summarized how strongly selected nodes remained connected within those same network contexts.

These findings also relate to recent work using electroencephalography (EEG) graph analysis to examine vulnerability to sleep deprivation. For example, Mason et al. [55] reported that EEG-derived network metrics were significantly associated with PVT impairment during sleep deprivation. Although EEG and rs-fMRI capture different aspects of brain dynamics, with EEG reflecting electrophysiological activity and rs-fMRI reflecting hemodynamic connectivity, both approaches converge on the idea that individual differences in vulnerability or resilience to sleep loss are reflected in large-scale network organization rather than isolated regional effects. In this context, the present results extend prior electrophysiological findings by localizing resilience-related network stability within thalamocortical and perceptual-memory subnetworks observed during ongoing sleep deprivation.

These findings connect existing models of cognitive resilience to specific network mechanisms. Cognitive resilience has been described as maintaining performance under stress or fatigue, with neural resilience reflecting stable functional architecture during such challenges [6]. Here, we identify the thalamocortical and perceptual-memory subnetworks whose preservation closely tracks variability in individual performance during sleep deprivation. These functional results are consistent with prior structural evidence: greater frontoparietal white-matter integrity predicted resistance to cognitive decline after TSD [20], and higher white-matter compactness within the DMN and SN predicted resistance to mood degradation during sleep loss [33]. This link between network stability and sustained behavior suggests candidate neural correlates of resilience expressed during ongoing sleep deprivation. Rather than serving as predictive biomarkers identified before sleep loss begins, these patterns may be more appropriately viewed as state-dependent markers that may be useful for future studies of fatigue-risk monitoring and performance preservation, including the development of targeted countermeasures such as optimized scheduling [26], pharmacological approaches [56], or neuromodulation [57].

Our findings should be interpreted in the context of prior studies that used baseline cognitive or neural measures to predict later vulnerability to sleep loss. Those approaches ask whether resilience can be anticipated before deprivation begins, whereas the present study addresses a different question: how neural network organization changes during ongoing sleep deprivation in relation to behavioral resilience. In that sense, baseline predictors and during-deprivation neural correlates address related but distinct questions and are best viewed as complementary rather than interchangeable.

Our design supports these interpretations. Collecting repeated resting-state fMRI scans across prolonged wakefulness is logistically demanding and therefore relatively uncommon in sleep deprivation research. The present dataset includes six usable imaging sessions spanning approximately 32 hours of continuous wakefulness, allowing us to track progressive network changes within individuals rather than relying on only two or three time points. This dense longitudinal sampling improves sensitivity to gradual connectivity shifts that accompany accumulating sleep pressure. In addition, imaging sessions were conducted within a consistent interval of the behavioral assessments, allowing us to relate network trajectories to vigilance performance within the temporal constraints of the protocol, as discussed in the Limitations. Mixed-effects modeling enabled us to characterize within-subject changes across prolonged wakefulness while accounting for inter-individual variability in behavioral performance [58]. The follow-up nodal strength analysis was intentionally restricted to ROIs implicated by the primary NBS results in order to provide node-level characterization of those subnetworks, and should therefore be interpreted as complementary rather than independent confirmatory evidence. Because these ROIs were selected on the basis of the observed NBS results rather than predefined hypotheses, this restriction should not be interpreted as an a priori reduction of the multiple-testing burden, but rather as a post hoc characterization of the same data-defined subnetworks.

Overall, resilience during prolonged wakefulness was associated with preserved thalamic and posterior cortical connectivity. These network dynamics may contribute to better maintenance of cognitive performance under rising homeostatic and circadian demands and may have implications for future fatigue-risk management strategies.

### Limitations

Several limitations of the present study should be acknowledged. First, our sine and cosine terms are based on clock time and approximate circadian phase only. They do not capture individual differences in circadian timing. Direct physiological phase markers such as dim light melatonin onset (DLMO) or core body temperature were not collected, so residual circadian influences may remain. Future studies should incorporate these measures and more granular behavioral sampling to better disentangle circadian from homeostatic contributions. Second, an a priori statistical power analysis was not conducted for the present study, and therefore it is not possible to determine whether the sample size was sufficient to detect all effects of interest. Although the repeated-measures longitudinal design increases sensitivity to within-subject changes across prolonged wakefulness, the adequacy of the sample size for detecting effects of a given magnitude cannot be established retrospectively. Future studies with larger cohorts and prospectively planned power analyses will be important for confirming the reproducibility and stability of the identified network patterns. Third, the PVT was administered approximately 2.5 hours after each fMRI session rather than immediately adjacent to scanning. Biomathematical models of fatigue and vigilance indicate that sustained-attention performance during continuous wakefulness changes over hourly timescales as a joint function of accumulated sleep pressure and circadian phase [59, 60]. These models do not provide a single universal estimate directly transferable to the reaction-speed units used for RSDM, because the expected magnitude of change depends on circadian timing, prior wakefulness, individual vulnerability, and the specific performance metric modeled. Nevertheless, the delay is likely to be most consequential for sessions occurring near the wake-maintenance zone (WMZ), habitual bedtime, or the circadian trough, where PVT performance can change meaningfully within a 2–3-hour window [21, 61, 62]. Moreover, functional network organization may itself vary over this timescale [62], meaning that the neural state at the time of scanning and the behavioral state captured by the subsequent PVT may reflect slightly different levels of accumulated sleep pressure and circadian phase. RSDM was derived as a between-session ratio of mean reaction speed rather than a point-in-time measure, which may provide some robustness to this source of variability; however, this does not fully mitigate the concern. This limitation is shared by many dense longitudinal fMRI studies of sleep deprivation given practical constraints of MRI scheduling, and future studies should aim to minimize the interval between imaging and behavioral assessment where feasible.

## Conclusion

In summary, NBS-defined subnetworks involving thalamic and perceptual-memory-related regions were associated with cognitive resilience during acute sleep deprivation. Using NBS as the primary analysis and nodal strength as a secondary node-level follow-up, we identified network patterns associated with individual differences in resilience during prolonged wakefulness. Based on six fMRI sessions obtained during prolonged wakefulness, preserved thalamocortical and posterior cortical connectivity may have implications for fatigue-risk monitoring, performance preservation, and targeted countermeasures.

## Supplementary Material

SupplementaryFigure_FINAL_01_zsag201

## Data Availability

Due to participant confidentiality and institutional data use agreements, the raw neuroimaging and behavioral datasets are not publicly available. Group-level summary data and statistical outputs that support the findings of this study are available from the corresponding author upon reasonable request and under a formal data use agreement approved by the University of Arizona. The analysis scripts used for network-based and nodal strength analyses are available from the corresponding author on request.
