# Supplementary material for: Functional brain network stability reflects individual resilience during sleep deprivation
Source: Sleep. 2026 Jul 21;49(10):zsag201. doi: 10.1093/sleep/zsag201 (PMC13658277; doi:10.1093/sleep/zsag201)
Supplement: Supplementary file 1 [file supplementaryfigure_final_01_zsag201.docx]

**Functional Brain Network Stability Reflects Individual Resilience during Sleep Deprivation**

Jungwon Cha^a^, David Negelspach^a^, Alisa Huskey^a^, Kathryn E. R. Kennedy^a^, Jason Katz^a^, Daniel B. Forger^b,c^ & William D. S. Killgore^a^
^a^Social, Cognitive, & Affective Neuroscience (SCAN) Laboratory, Department of Psychiatry, University of Arizona College of Medicine, 1501 N. Campbell Avenue, Tucson, AZ, USA

^b^Department of Mathematics, University of Michigan, Ann Arbor, MI, USA

^c^Gilbert S. Omenn Department of Computational Medicine and Bioinformatics, University of Michigan, Ann Arbor, MI, USA

Email address: Jungwon Cha (jungcha@arizona.edu), David Negelspach (davidnegelspach@arizona.edu), Alisa Huskey (ahuskey@arizona.edu), Kathryn E. R. Kennedy (katkennedy@arizona.edu), Jason Katz (jasonkatz2721@arizona.edu), Daniel Forger (forger@umich.edu) & William D. S. Killgore (killgore@arizona.edu)

Corresponding author: Jungwon Cha,

Department of Psychiatry,

University of Arizona College of Medicine

1501 N. Campbell Avenue, Tucson, AZ 85724, USA

Email: jungcha@arizona.edu

**Figure S1. PVT reaction speed trajectories by resilience group.**
PVT reaction speed (1000/RT) across hours awake is shown for participants stratified into High and Low RSDM groups (top and bottom 40%). Points represent group means and error bars indicate SEM. Higher-RSDM participants showed less steep declines in performance across prolonged wakefulness. These visualizations are descriptive only; all statistical analyses treated RSDM as a continuous variable. The modest rebound at later time points may reflect circadian influences rather than recovery.

**Figure S2. Full interaction-statistic matrix for the RSDM × Hours Awake interaction.** Shown is the full edge-wise matrix of unthresholded t-statistics for the RSDM × Hours Awake interaction across all tested ROI-to-ROI functional connections. ROIs are grouped anatomically as left subcortical, right subcortical, left cortex, and right cortex. Edges belonging to the NBS-defined thalamocortical subnetwork are overlaid as magenta squares, and edges belonging to the perceptual-memory subnetwork are overlaid as cyan circles. This figure is provided to display the significant subnetworks in the context of the full unthresholded interaction-statistic matrix. Formal statistical inference is based on the mixed-effects models and permutation-based NBS analyses reported in the main text.
